# Norepinephrine is More Effective Than Midodrine/Octreotide in Patients With Hepatorenal Syndrome-Acute Kidney Injury: A Randomized Controlled Trial

**DOI:** 10.3389/fphar.2021.675948

**Published:** 2021-07-02

**Authors:** Eman Ibrahim El-Desoki Mahmoud, Doaa H Abdelaziz, Sherief Abd-Elsalam, Noha O. Mansour

**Affiliations:** ^1^ Intensive Care Medicine, National Hepatology and Tropical Medicine Research Institute, Cairo, Egypt; ^2^ Department of Clinical Pharmacy, The National Hepatology and Tropical Medicine Research Institute, Cairo, Egypt; ^3^ Pharmacy Practice and Clinical Pharmacy Department, Faculty of Pharmacy, Future University in Egypt, Cairo, Egypt; ^4^ Department of Tropical Medicine and Infectious Diseases, Faculty of Medicine, Tanta University, Tanta, Egypt; ^5^ Clinical Pharmacy and Pharmacy Practice Department, Faculty of Pharmacy, Mansoura University, Mansoura, Egypt

**Keywords:** norepinephrine, midodrine /octreotide, hepatorenal syndrome, acute kidney injury, intensive care, cirrhosis, renal impairment

## Abstract

**Background:** Terlipressin is the first-line pharmacological treatment for hepatorenal syndrome. When terlipressin is unavailable, midodrine/octreotide or norepinephrine, with albumin, represent the alternative treatments. The comparative efficacy of these alternative regimens remains unclear.

**Objective:** To compare the efficacy of midodrine/octreotide to that of norepinephrine for the treatment of patients with hepatorenal syndrome.

**Methods:** In the intensive care setting, sixty patients with hepatorenal syndrome were randomized to initially receive either 0.5 mg/h of norepinephrine (maximum 3 mg/h) or 5 mg of oral midodrine three times/day (maximum 12.5 mg three times/day) plus octreotide (100 μg/6 h) as subcutaneous injection (maximum 200 μg/6 h), together with albumin (20–40 g/day). Treatment was allowed for a maximum of 10 days. Survival was analyzed for up to 30 days. The primary efficacy outcome was the proportion of patients who achieved full response, defined as the return of serum creatinine to a value within 0.3 mg/dl of the baseline at the end of treatment.

**Results:** There was a significantly higher rate of full response in the norepinephrine group (15/26, 57.60%) than the midodrine/octreotide group (5/25, 20%) (*p* = 0.006). Eleven (42.30%) patients in the norepinephrine group and 6 (24%) in the midodrine/octreotide group survived (*p* = 0.166).

**Conclusion:** Norepinephrine plus albumin is significantly more effective than midodrine and octreotide plus albumin in improving renal function in patients with hepatorenal syndrome.

(ClinicalTrials.gov, identifier: NCT03455322).

https://clinicaltrials.gov/ct2/show/NCT03455322?cond = Hepatorenal+Syndrome&cntry = EG&draw = 2&rank = 1.

## Introduction

Hepatorenal syndrome (HRS) is defined as renal failure occurring in patients with cirrhosis and ascites in the absence of other causes of renal dysfunction (Israelsen et al., 2017). Patients with HRS have marked circulatory dysfunction with splanchnic arterial vasodilatation, resulting in severe renal vasoconstriction (Wong and Blendis., 2001). HRS-acute kidney injury (HRS-AKI)—known formerly as HRS type 1—is often fatal, resulting in the death of almost 50% of patients within 2 weeks of diagnosis (Alessandria et al., 2005). The only definitive treatment for eligible patients with HRS-AKI is liver transplantation (Ginès et al., 2003). The role of the transjugular intrahepatic porto-systemic shunt (TIPS) in the management of HRS remains controversial. However, recent data have suggested that the TIPS might have better renal outcomes, with high incidence of post-TIPS hepatic encephalopathy (Song et al., 2018). The currently available pharmacological option for HRS-AKI management is the administration of systemic vasoconstrictors with albumin. This approach has been found to be an effective option that ameliorates renal dysfunction and improves survival (Wang et al., 2018). Selection of the medical therapy for patients with HRS-AKI depends upon whether the patient is critically ill and the availability of certain medications, which varies regionally. According to the latest European Association for the Study of the Liver (EASL) practice guidelines, the first-line pharmacological treatment for HRS-AKI is terlipressin in combination with albumin (The European Association for the study of the Liver, 2018; Nanda et al., 2018); however, this vasopressin analog is not available in many countries, including the United States (Singh et al., 2012). The high cost of terlipressin therapy might represent another major obstacle, particularly in developing countries (Wechowski et al., 2007). Accordingly, several attempts have been made recently to obtain alternative effective strategies for improving renal functions and extending the patients’ survival before transplantation. Vasoconstrictors other than vasopressin analogs that have been used in the management of HRS-AKI include norepinephrine and midodrine plus octreotide, both in combination with albumin. Review of recent evidence has shown that the widely available inexpensive norepinephrine has a similar efficacy to terlipressin (Wang et al., 2018; Zhang et al., 2019); however, it always requires a central venous line and the transfer of the patient to an intensive care unit (ICU) (Runyon, 2009). The midodrine and octreotide combination, along with albumin infusion, represents another alternative that has been reported from Europe and the United States (Runyon, 2009). This regimen can be administered outside an ICU and can even be given at home. The comparative efficacy of midodrine plus octreotide vs. norepinephrine in the management of HRS-AKI remains unclear, and the data available are of low-quality evidence. Only one pilot study with less than ten HRS-AKI patients in each arm deduced that norepinephrine has comparable efficacy with midodrine plus octreotide (Tavakkoli et al., 2012). Further investigations using the most up-to-date HRS diagnostic criteria are warranted. Therefore, this study aimed to investigate the efficacy of norepinephrine vs*.* midodrine plus octreotide and to determine the predictive factors of response in patients with HRS-AKI.

## Materials and Methods

This was a parallel-group, open-label, randomized controlled study (ClinicalTrials.gov registration number: NCT03455322). The protocol was approved by the Institutional Review Board of the National Hepatology and Tropical Medicine Research Institute (NHTMRI), Cairo, Egypt. All study procedures were performed in accordance with good clinical practice and the Declaration of Helsinki. Written informed consent was obtained from each patient or a legally authorized representative prior to enrollment in the study.

### Patients

Both sexes aged 18 years or older having cirrhosis, ascites, and a diagnosis of HRS-AKI based on the 2015 International Club of Ascites (ICA) diagnostic criteria (Angeli et al., 2015) were eligible for participation. Exclusion criteria included serum creatinine (sCr) > 7 mg/dl, hypotension (mean arterial pressure (MAP) < 70 mm Hg), or sepsis. Other exclusion criteria included recent treatment with nephrotoxic drugs or vasopressors. Patients with severe cardiovascular disease, advanced hepatocellular carcinoma, or a known allergy to the study medications were also excluded.

### Study Procedures and Treatment Regimens

Screening and eligibility criteria were verified upon admission to the ICU. A diagnosis of AKI was established through comparing the sCr value at the time of ICU admission to that obtained from the patient’s records, which was defined as the lowest and the most recent sCr measurement obtained within the previous 3 months (Angeli et al., 2015). Patients who met all other diagnostic criteria of HRS-AKI provided by the previous definition (Angeli et al., 2015) were enrolled in the study. Qualified patients were subjected to baseline assessments after signing informed consent forms. It included vital sign measurements, presence of comorbidities, height and weight, the Child–Pugh score, and the sequential organ failure assessment (SOFA) score. Vital signs, Blood urea nitrogen, sCr, serum sodium, serum albumin, total bilirubin, urine output, and complete blood count with differential were measured at the baseline and on a daily basis throughout the study period. We generated the two comparison groups using simple randomization, with a 1:1 allocation ratio, by using a computer random sequence generator. Patients received either a continuous infusion of norepinephrine at an initial dose of 0.5 mg/h (maximum 3 mg/h) or 5 mg of oral midodrine three times/day (maximum 12.5 mg three times/day) plus octreotide (100 μg/6 h) as subcutaneous injection (maximum 200 μg/6 h). Treatment allocation was concealed from outcome assessors and patients using sequentially numbered, opaque, sealed envelopes kept by the hospital pharmacist. The envelopes were opened sequentially only after participant details had been written on the envelope. The principal investigator enrolled participants and assigned them to interventions. The duration of treatment was allowed to extend to a maximum of 10 days. The administration of albumin at doses of 20–40 g/day was recommended, as clinically indicated, for all patients in both study arms as per current ICA guidelines (Angeli et al., 2015). Patients or their first-degree relatives were contacted 30 days after the first day of study treatment for assessment of survival through phone calls. Survival was analyzed for up to 30 days and was defined as the time (in days) that each patient survived from the day of randomization; death was included as an event. No clinical laboratory tests were scheduled to occur during the follow-up period. Data of patients who died within 2 days of randomization were excluded from the final analysis.

### Outcome Assessment

#### Efficacy Outcomes

The primary efficacy outcome was the proportion of patients who achieved full response, defined as the return of sCr to a value within 0.3 mg/dl of the baseline at the end of treatment (Angeli et al., 2015). Secondary outcomes included the proportion of patients who achieved partial response, defined as a regression of at least one AKI stage, with a fall in the sCr value to ≥0.3 mg/dl above the baseline value (Angeli et al., 2015). Incidence of HRS reversal, defined as at least one sCr value of ≤1.5 mg/dl while on treatment (Boyer Thomas et al., 2012), change in renal functions from the baseline through the end of treatment, incidence of HRS-AKI relapse 30 days after cessation of treatment, and overall survival through 30 days after randomization were assessed as secondary outcomes.

#### Safety Outcomes

Safety outcome measures included recording adverse events experienced throughout the study period in both treatment groups. The incidence of hepatic encephalopathy episodes, bacterial infections, gastrointestinal bleeding, myocardial infarction, arrhythmia, and arterial hypertension was assessed at the end of the study. The need for mechanical ventilation and the need for dialysis were determined throughout the treatment period.

### Sample Size Calculations

The primary end point of the study was full response at the end of treatment, and this was used for sample size calculation. It was hypothesized that complete recovery of renal function could occur in 70% of patients treated with norepinephrine and in 24% of those treated with midodrine plus octreotide (Cavallin et al., 2015; Nanda et al., 2018). Using a two-tailed test, 22 patients were required in each group, for a *p* value < 0.05 with an *a* error of 5% and a *ß* error of 20%. To compensate for the lost follow up, the sample size was increased to 30 in each group.

### Statistical Analysis

A per-protocol analysis was used to analyze the data. Numerical data were expressed as mean and standard deviation or median and interquartile range as appropriate. Qualitative data were expressed as frequency and percentage. Numerical data were tested for normality using the Shapiro–Wilk test. Data were found to be not normally distributed, so the nonparametric tests were used. Comparison between the two groups was carried out using the Mann–Whitney test. The Wilcoxon-signed ranks test was used to compare two consecutive measures of numerical variables. For categorical data, the chi-square test was applied. The results were analyzed at the baseline and on day 10 of the study. Survival curves were calculated using the Kaplan–Meier method and compared using the log-rank test. Factors predictive of response to therapy were assessed by univariate analysis using the Mann–Whitney test for continuous data and the chi-square test for categorical data. Due to the small numbers of partial responders recorded in the current study (one patient in the norepinephrine group and one in the midodrine/octreotide group), they were added to the nonresponder group during analysis. Multivariate analysis was performed for significant predictors of response. A value of *p* < 0.05 was taken as significant.

## Results

**FIGURE 1:**
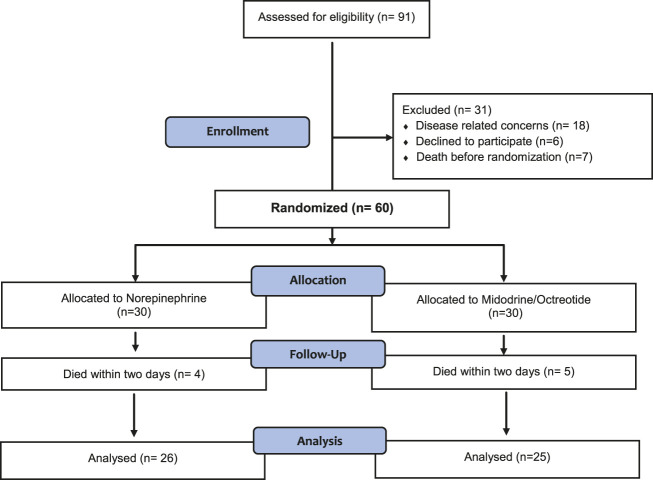
CONSORT flow diagram showing the flow of patients throughout the study.

As shown in Figure 1, eighty five percent (*n* = 51) of the randomized subjects completed the study and were included in the final analysis.

### Baseline Characteristics

The baseline demographic profile and clinical and laboratory parameters were similar in patients receiving norepinephrine and midodrine plus octreotide (Table 1). The etiology of liver cirrhosis was the hepatitis C virus (HCV) in 54 patients (90%) and the hepatitis B virus (HBV) in six patients (10%) in both groups. In the norepinephrine group, responders received a median dose which equals to 1.08 mg/h (range 0.5–3.0 mg/h) for a median duration of 9 days (range 3–10 days). In the midodrine/octreotide group, responders were given a median dose of midodrine which equals to 7.5 mg/day (range 5–12.5 mg/day) for a median duration of 10 days (range 3–10 days). There was no significant difference between the mean dose of albumin (26.33 ± 5.6 g/day) in the norepinephrine group and that (29.33 ± 7.4 g/day) in the midodrine/octreotide one (*p* = 0.11). The median albumin doses are the same in both groups (30 g/day).

**TABLE 1:** Baseline demographic, clinical, and biochemical variables of the study population.

	Midodrine/octreotide (*n* = 30)	Norepinephrine (*n* = 30)	*p* value
Age (years)	61.85 ± 8.21	59.92 ± 7.45	0.705
Weight (kg)	74.40 ± 9.33	78.84 ± 10.62	0.226
BMI (kg/m2)	26.74 ± 3.32	29.01 ± 4.11	0.115
Gender; *n* (%)	—	—	0.121
Male	12 (40)	18 (60)	—
Female	18 (60)	12 (40)	—
Comorbidities; *n* (%)	—	—	—
Diabetes mellitus	10 (33.33)	14 (46.66)	0.292
Hypertension	8 (26.66)	9 (30)	0.774
Child–Pugh category	—	—	0.688
B n (%)	3 (10)	4 (13.3)	—
C n (%)	27 (90)	26 (86.7)	—
Child–Pugh score	12.13 ± 1.90	11.37 ± 1.99	0.157
SOFA score	8.90 ± 2.98	6.96 ± 1.96	0.072
Pulse	84.70 ± 18.95	86.20 ± 13.26	0.492
CVP*	14.00 (4.00)	13.00 (3.00)	0.270
Mean arterial pressure (mmHg)	77.00 ± 7.13	77.66 ± 8.66	0.743
Hemoglobin (gm%)	9.64 ± 2.14	9.91 ± 1.67	0.679
White blood cells (mm3)	10.00 ± 3.41	11.33 ± 6.41	0.762
Platelets (mm3)*	108.50 (56.26)	101.0 (91.00)	0.267
Serum bilirubin (mg/dl) *	3.95 (8.65)	3.500 (6.55)	0.605
Serum albumin (g/dl)	2.349 ± 0.414	2.481 ± 0.333	0.094
Serum sodium (mEq/L)	129.95 ± 9.849	125.24 ± 9.161	0.428
Blood urea nitrogen (mg/dl)*	152.30 (73.50)	148.1 (101.8)	0.478
Serum creatinine (mg/dl)	2.466 ± 0.872	2.72 ± 0.905	0.187
Urine output (ml/day)*	405 (445.00)	420 (457.50)	0.647

Data are mean ± SD; *Median (IQR); SOFA, sequential organ failure assessment score; CVP, central venous pressure; *n*, number of patients.

### Efficacy Outcomes

Table 2 shows the effect of norepinephrine compared to that of midodrine plus octreotide on different parameters at the baseline and on day 10 of the study. After treatment, 15 (57.60%) patients in the norepinephrine group and 5 (20%) in the midodrine/octreotide group responded to therapy (*p* = 0.006). A marked decrease in sCr from the baseline was observed at the end of the study in the norepinephrine group (*p* < 0.05). In the same treatment group, the urine output and the MAP increased significantly at day 10 (*p* < 0.05). On the contrary, the sCr and the MAP were deemed comparable to baseline levels in patients assigned to the midodrine/octreotide group; trends toward increase in urine output were shown at the end of the study, but the difference was not statistically significant (*p* > 0.05). After 30 days, none of the responded patients in either group experienced relapse of HRS.

**TABLE 2:** Change in parameters with therapy in the two study groups.

Parameter	Midodrine/octreotide (*n* = 25)			Norepinephrine (*n* = 26)		
	Baseline	End of treatment	*p* value	Baseline	End of treatment	*p* value
Serum creatinine (mg/dl)	2.570 ± 0.820	2.512 ± 1.07	0.447^a^	2.723 ± 0.846	2.246 ± 1.170	0.037^a^
Urine output (ml/day)	627.3 ± 469.5	699.0 ± 611.5	0.648^a^	545.3 ± 432.6	943.8 ± 925.4	0.022^a^
Mean arterial pressure (mmHg)	76.52 ± 5.91	76.50 ± 17.47	0.922^a^	78.65 ± 8.87	84.79 ± 11.35	0.018^a^
Need for dialysis; *n* (%)	------	4 (16.00)	—	------	0 (0)	—
Need for tapping; *n* (%)	------	12 (48.00)	—	------	14 (53.84)	0.676^b^
Amount tapped (L)	—	7.58 ± 3.67	—	—	5.07 ± 2.272	0.116^b^
HRS reversal; *n* (%)	------	11 (44)	—	------	13 (50)	0.668^b^
Responders; *n* (%)	------	5 (20.00)	—	------	15 (57.60)	0.006^b^
Surviving patients; *n* (%)	—	6 (24.00)	—	—	11 (42.30)	0.166^b^

Data are mean ± SD; *n*, number of patients.

a Comparison between data at the baseline and the end of treatment.

b Comparison between the midodrine/octreotide group and the norepinephrine group at the end of treatment.

### Safety Outcomes

No significant difference was found regarding the serious adverse events experienced in both groups, as summarized in Table 3, although a higher number of patients were detected in the midodrine/octreotide combination group. In the norepinephrine group, two patients experienced atypical chest pain with normal electrocardiograms. Three patients developed dyspnea in the midodrine/octreotide combination group. These adverse events were self-limiting. Pulmonary edema was observed in two patients (one in each group) who were mechanically ventilated. Myocardial infarction, arrhythmia, and arterial hypertension were not observed in any of the patients in either group.

**TABLE 3:** Serious adverse events experienced in both groups.

	Midodrine/octreotide	Norepinephrine	*p* value
Hepatic encephalopathy	12 (48)	7 (26.9)	0.120
Need for mechanical ventilation^a^	12 (48)	7 (26.9)	0.120
Infection	14 (56)	8 (30.7)	0.069
Gastrointestinal bleeding	5 (20)	5 (19.2)	0.945

a Causes of mechanical ventilation include shock, sepsis, hepatic encephalopathy, pulmonary embolism, pulmonary edema, and multi-organ dysfunction.

### Survival Analysis

**FIGURE 2:**
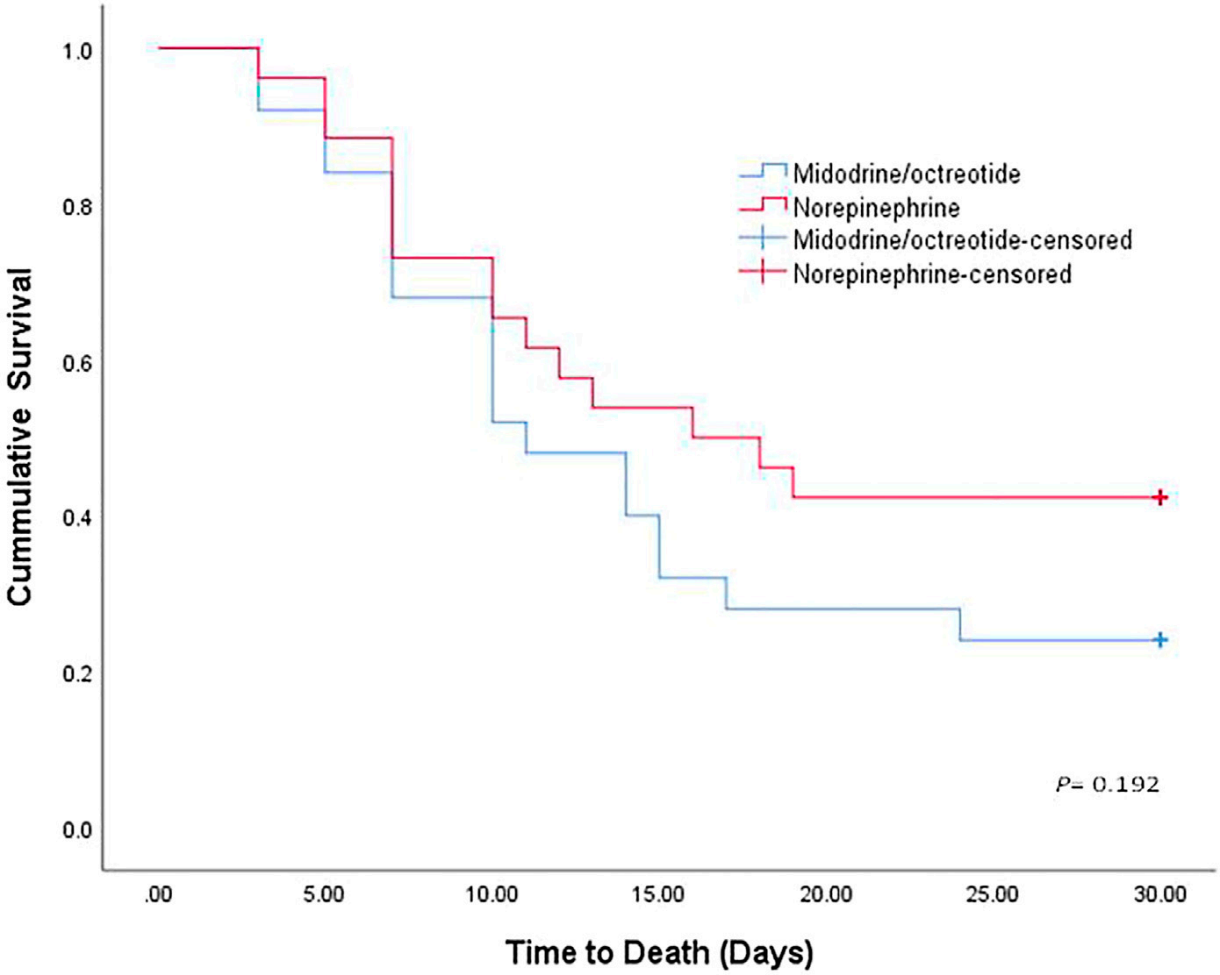
Kaplan–Meier curve showing the cumulative probability of overall survival of patients treated with norepinephrine and midodrine plus octreotide.

Eleven (42.30%) patients in the norepinephrine group (nine responders and two nonresponders) and 6 (24%) in the midodrine/octreotide group (four responders and two nonresponders) survived (*p* = 0.166). The median survival time was 16 days with norepinephrine and 11 days with midodrine/octreotide. The survival curves shown using the Kaplan–Meier method (Figure 2) were compared using the log-rank test and were not statistically different in the two groups (*p* = 0.192). In-hospital mortality was documented in a single patient who responded to midodrine/octreotide treatment. This patient experienced cardiac arrest due to pulmonary embolism. In the norepinephrine group, in-hospital mortality was observed in three responders, who died as a result of septic shock after worsening of hepatic encephalopathy, whereas mortality after patient discharge was recorded for three other responders. Causes of mortality were unknown for these three patients. Mechanical ventilation preceding mortality was needed for all responders during their hospital stay. Worsening of renal functions was the leading cause of in-hospital mortality in nonresponders.

### Predictors of Response

Several variables obtained at the baseline were analyzed for the predictive value of response to treatment. Univariate analysis (Table 4) showed that lower baseline values of the Child–Pugh score and the SOFA score were associated with response. However, none of them was found to be an independent predictor of response in the multivariate analysis (Supplementary Table S1).

**TABLE 4:** Univariate analysis of baseline variables according to response to treatment.

Variables	Responders (*n* = 20)	Nonresponders† (*n* = 31)	*p* value
Gender (M:F)	14:6	14:17	0.082
Assigned treatment; *n* (%)	—	—	0.006
Midodrine/octreotide	5 (25)	20 (64.52)	—
Norepinephrine	15 (75)	11 (35.48)	—
Age (year)	58.60 ± 8.39	61.60 ± 6.98	0.143
Serum bilirubin (mg/dl)*	3.12 (8.10)	3.0 (5.60)	0.132
Serum albumin (g/dl)	2.38 ± 0.457	2.32 ± 0.454	0.961
Child–Pugh score	10.85 ± 2.007	12.11 ± 1.77	0.031
Serum sodium (mEq/L)	125.5 ± 7.93	128.7 ± 8.40	0.275
Serum creatinine (mg/dl)	2.925 ± 0.981	2.471 ± 0.670	0.06
Blood urea nitrogen (mg/dl)	149.4 ± 70.53	148.6 ± 53.89	0.893
Urine output (ml/day)	652.5 ± 454.9	563.2 ± 453.7	0.410
MAP (mmHg)	77.20 ± 7.585	77.89 ± 7.99	0.567
SOFA score	6.30 ± 2.17	7.785 ± 2.92	0.045

Data are mean ± SD; *Median (IQR); MAP, mean arterial pressure (mmHg); SOFA, sequential organ failure assessment score; *n*, number of patients. † Two patients were considered as partial responders (one patient in each study group)^12^.

## Discussion

Hepatorenal syndrome is the most aggressive complication of portal hypertension and is associated with poor survival, measured in weeks. Vasoconstrictors have shown promising results in patients with HRS by causing splanchnic vasoconstriction and increasing effective arterial blood volume (Nanda et al., 2018). In the present study, we evaluated the role of norepinephrine vs*.* midodrine/octreotide, plus albumin, in the management of HRS-AKI in cirrhotic patients. In the norepinephrine group, reversal of HRS was seen in 50% (*n* = 13) and full response was documented in 57% (*n* = 15) of patients. The rate of reversal presented in our study was considerably lower than the previous reported rates for norepinephrine in reversing HRS. Duvoux reported reversal of HRS in 10 out of 12 (83%) patients (Duvoux et al., 2002). Alessandria reported reversal in three out of 4 (75%) patients (Alessandria et al., 2007). The small sample size enrolled in these studies (Duvoux et al., 2002; Alessandria et al., 2007) and the inclusion of patients (60%) diagnosed with non-HRS AKI in the first study (Duvoux et al., 2002), which is characterized by better prognosis and higher incidence of renal recovery than HRS-AKI, might explain the lack of connection with our results.

However, response rates observed in the current study (57.6%) were considered slightly higher than those found by Sharma, who showed a 40% response with norepinephrine (Sharma et al., 2008). The same finding was seen in a study by Singh, in which 43.4% of patients responded to norepinephrine (Singh et al., 2012). These results suggested that norepinephrine was effective in improving renal functions in patients with HRS-AKI, as explained in the current study by the improvement of circulatory functions, reflected through a significant increase in urine output and MAP on day 10. Differences in response rates among studies might be attributed to variations in defining response to therapy. The primary outcome of the current study was adopted from the 2015 ICA consensus (the most recent consensus at the time of study commencement), which depends on new definitions of AKI to explain patients with full response, while the previously reported studies (Duvoux et al., 2002; Alessandria et al., 2007; Sharma et al., 2008; Singh et al., 2012) used HRS reversal as their main outcome. Although a positive impact of the new definition on the rate of response to the pharmacological treatment is expected, it should be confirmed by further studies.

Regarding the midodrine/octreotide group, 20% of the patients (*n* = 5) completely responded to the treatment. The rate of full response to midodrine/octreotide was similar to that (20.8%) reported by Cavallin, who compared the efficacy of terlipressin to that of midodrine/octreotide in a randomized controlled trial (Cavallin et al., 2015). Nevertheless, it was lower than the response (40%) observed in a previous retrospective study by Esrailian et al. (2007). In order to explain this difference, it should be highlighted that more than 50% of the treated patients in the study by Esrailian (Esrailian et al., 2007) had alcoholic hepatitis, in which recovery of renal functions could have been achieved only as a result of the improvement of their liver disease. Besides, in the same study, several patients were co-treated with pentoxyphylline, and the treatment with midodrine plus octreotide was not found to be an independent predictor of renal response.

Considering the comparison between norepinephrine and midodrine/octreotide, there was a statistically significant difference in complete response rates between the two regimens at the end of the study, which showed a favorable efficacy of the norepinephrine plus albumin regimen. To the best of our knowledge, there is poor-quality evidence from a single pilot trial regarding the comparative efficacy of these two regimens. Nonetheless, the meta-analysis of randomized trials that reported equal efficacy of terlipressin and norepinephrine (Mattos et al., 2016) and the previously reported superior efficacy of terlipressin compared to the midodrine/octreotide combination (Cavallin et al., 2015) might support the superior efficacy of norepinephrine compared to the midodrine plus octreotide regimen in improving renal outcomes in HRS-AKI patients. On the other hand, our result opposed the finding of the pilot study conducted by Tavakkoli, who reported a similar efficacy of norepinephrine to that of midodrine/octreotide in the induction of complete response in HRS patients (Tavakkoli et al., 2012). The small sample size enrolled in Tavakkoli’s study, the longer treatment duration (15 days), and the inclusion of a considerable percentage of patients (45.5%) with non-HRS AKI might explain the lack of correlation with our results.

Various predictive factors of response (gender, baseline Child–Pugh score, serum bilirubin, sCr, MAP, and urine output) have been reported in previous studies on patients with HRS (Colle et al., 2002; Nazar et al., 2010; Boyer T. D. et al., 2011). In the present study, the univariate analysis showed that lower values of the baseline Child–Pugh score and the SOFA score were associated with response. However, in multivariate analysis, none of them was found to be an independent predictor of response. This finding was not supported by the results of previous studies, which found that a higher sCr value at the beginning of treatment leads to a lower probability of response (Boyer T. D. et al., 2011; Rodríguez et al., 2014). However, because our findings depend upon the 2015 ICA consensus that omitted the final cutoff value of sCr in defining HRS-AKI, clinicians should be encouraged to initiate vasoconstrictors at the time of diagnosis and irrespective of the magnitude of change in sCr.

The difference in survival rate between the two study groups was insignificant, however; it was lower in those assigned to the midodrine/octreotide group. Eleven (42.30%) patients in the norepinephrine group (nine responders and two nonresponders) and 6 (24%) in the midodrine/octreotide group (four responders and two nonresponders) survived at 30 days. Low survival rates documented in the present study are comparable with others’ findings and reported even with terlipressin [15.7% (Srivastava et al., 2015) and 27% (Martín-Llahí et al., 2008)].

An insignificant improvement in survival rates between different treatment regimens despite marked progress in renal outcomes was previously reported (Martín-Llahí et al., 2008; Sanyal et al., 2008; Cavallin et al., 2015; Srivastava et al., 2015). Moreover, a lower survival rate (20%) was reported among patients who received norepinephrine despite significant difference in response (Arora et al., 2020). Lower survival rates in responders might be attributed to the advanced stages of liver disease observed in the current study (>85% Child–Pugh C), as patients may nonetheless continue to have other complications of decompensated cirrhosis that are unrelated to HRS-AKI and die from these complications despite using vasoconstrictors that improve their kidney functions. This confirms the survival benefits of liver transplantation as the definitive HRS treatment. The treatment with either regimen was well tolerated, and only two patients in the norepinephrine group developed mild, self-limiting, atypical chest pain with normal electrocardiograms. This finding was consistent with that observed by Sharma et al. (Sharma et al., 2008), who reported reversible ST depression in one patient on norepinephrine infusion. This suggested that norepinephrine is a safe and tolerable treatment option that improves hemodynamics in patients with HRS-AKI. The incidence of pulmonary edema was small in both groups. Although respiratory failure due to pulmonary edema was recently reported with the use of vasoconstrictors (Wong et al., 2021) and albumin (China et al., 2021), it could not be linked in the current study to the use of any of the studied medications due to the small number of patients that suffered from this adverse event. Other reported adverse events were comparable in both groups, which might be attributed to further liver decompensation due to hepatitis-related cirrhosis.

One limitation of our study was conducting it in a single center, which might limit the generalization of our results. Bias cannot be excluded as the treatment arms were not blinded for the investigators. Another limitation was that all included patients were suffering from hepatitis-related cirrhosis, in which the outcomes of patients differ from those with cirrhosis due to other causes. Future research should focus on performing cost-effectiveness analysis, considering all direct and indirect medical expenditures before favoring norepinephrine in terms of cost.

In conclusion, the results of this study indicated that administration of norepinephrine plus albumin is more effective in improving renal functions in patients with HRS-AKI than the administration of midodrine and octreotide plus albumin. Thus, where terlipressin is unavailable, norepinephrine plus albumin should be considered the first choice for management of patients with HRS-AKI.

## Data Availability

The raw data supporting the conclusions of this article will be made available by the corresponding author on reasonable request.
