# Supplementary material for: Norepinephrine is More Effective Than Midodrine/Octreotide in Patients With Hepatorenal Syndrome-Acute Kidney Injury: A Randomized Controlled Trial
Source: Front Pharmacol. 2021 Jul 2;12:675948. doi: 10.3389/fphar.2021.675948 (PMC8283260; doi:10.3389/fphar.2021.675948)
Supplement: Supplementary file 1 [file Table1.docx]

**Supplementary data**

**S1: Multivariate analysis (including variables with p value <0.05 in univariate analysis).**

| Parameter | *P value* | 95% confidence interval | |
| --- | --- | --- | --- |
|  |  | Lower bound | Upper bound |
| Child-Pugh score | 0.173 | -0.272 | 0.050 |
| SOFA score | 0.332 | -0.179 | 0.062 |

SOFA: sequential organ failure assessment score
